# Royal Jelly and Its Dual Role in TNBS Colitis in Mice

**DOI:** 10.1155/2015/956235

**Published:** 2015-03-02

**Authors:** Luis Paulo Manzo, Felipe Meira de-Faria, Ricardo José Dunder, Eduardo Augusto Rabelo-Socca, Silvio Roberto Consonni, Ana Cristina Alves de Almeida, Alba Regina Monteiro Souza-Brito, Anderson Luiz-Ferreira

**Affiliations:** ^1^Department of Pharmacology, Faculty of Medical Sciences, University of Campinas (UNICAMP), 13083-970 Campinas, SP, Brazil; ^2^Department of Structural and Functional Biology, Institute of Biology, University of Campinas (UNICAMP), 13083-970 Campinas, SP, Brazil; ^3^Department of Biochemistry and Tissue Biology, Institute of Biology, University of Campinas (UNICAMP), 13083-970 Campinas, SP, Brazil; ^4^Department of Biological Sciences, Federal University of Goiás (UFG), Regional Catalão, 75704-020 Catalão, GO, Brazil

## Abstract

Royal Jelly (RJ) is widely consumed in diets throughout the world due to its beneficial effects: antioxidant, antitumor and anti-inflammatory. We have investigated the role of RJ in the development of TNBS colitis in mice. Colitis was induced by a rectal instillation of TNBS at 0.1 mL per mouse. Intestine samples of the animals orally treated with RJ (100, 150, and 200 mg/kg) were collected for antioxidant assays (GSH and GSH-Px), proinflammatory protein quantification (COX-2 and NF-*κ*B), and histological analyses. RJ 100 mg/kg maintained GSH levels and increased the activity of GSH-Px, downregulated key inflammatory mediators (COX-2 and NF-*κ*B), and decreased the lesions caused by TNBS as shown by the histological analyses. In conclusion, RJ showed anti-inflammatory and antioxidant properties in experimental colitis, resulting in the amelioration of the macroscopic and histological analyses. These results corroborate with the RJ supplementation in diets.

## 1. Introduction

Royal jelly (RJ) is an important functional food item that possesses several health-promoting properties and has been widely used in commercial medical products, healthy foods, and cosmetics in many countries [1]. The myriad effects accredited to RJ are comprehensible due to its richness in vitamins, minerals, proteins, amino acids, and carbohydrates [2]. Some of its accredited properties are immunomodulation [3] anti-inflammatory [4], healing [5], and antiaging [6]. It is a nourishing substance secreted by the mandibular and hypopharyngeal glands of worker honey bees* Apis mellifera* [7]. On one hand, royal jelly is accredited to exert many beneficial effects to the body; on the other, there are some reports of anaphylactic shock, allergy, and acute colitis due to its intake [8–10]. Recently, due to the biological effects accredited to RJ, its use has been increasing throughout the world, which, in turn, has been attracting enormous attention from the scientific community. It is already known that highly reactive free radicals are formed by either exogenous chemicals or endogenous metabolic processes of the body. These free radicals are capable of oxidizing biomolecules, resulting in tissue damage and consequent cell death [11] in various pathological processes including inflammatory bowel disease [12, 13].

Ulcerative colitis (UC) and Crohn's disease (CD) are the two major forms of inflammatory bowel diseases (IBD). Both UC and CD are of complex etiology and are believed to arise from the interaction of genetic, environmental, and microbial factors [14]. These two forms of IBD occur in the large intestine, although CD may affect the whole digestive tract while UC tends to be restricted to the distal portion of the tube (rectum) [15]. Either CD or UC presents ulcerated areas; the first shows a transmural lesion, affecting the entire wall thickness (from the mucosa to the serosa). The second is shallower, affecting the mucosal lining only. Many authors have proposed that these intestinal conditions are mediated by the activation of both lymphocytes and nonlymphoid cells such as macrophages and neutrophils. Once a vast number of these cells are activated, they migrate to the harmed mucosa site, leading to an overproduction of oxygen free radicals that may damage or even kill the cells in the inflamed area [16].

The antioxidant and anti-inflammatory mechanisms are of crucial importance to the homeostasis and integrity of the bowel. Since scientists have not developed an effective drug for the IBD treatment yet [9], new and alternative therapies arise not only as a promise but, rather, as a necessity for the patients undergoing such conditions. Due to the anti-inflammatory [4] and antioxidant properties of RJ [1], the oxidative stress taking place in the intestine of UC and CD patients, and a study reporting that RJ was effective at attenuating acetic acid-induced colitis in rats [17], our group decided to investigate whether an oral pretreatment with RJ would have any effect on mice submitted to TNBS-induced colitis.

## 2. Materials and Methods

### 2.1. Animals

Unib-SW 6–8-week-old female mice were purchased from CEMIB-UNICAMP. All the protocols used in this study were in accordance with the Ethics Committee on the Use of Experimental Animal (CEUA) from UNICAMP (2398-1). All animals had free access to tap filtered water and certified rodent chow (Nuvilab) during the whole experiment. Room temperature was kept constant with 12/12 light dark cycles 60 ± 1% humidity and a temperature of 21 ± 2°C.

### 2.2. Drugs, Reagents, and Royal Jelly

All drugs and reagents were prepared right before use and were of chemical grade. 2,4,6-Trinitrobenzene sulfonic acid (TNBS) and NaCl were bought from Sigma (MO, USA). Royal Jelly was obtained from Baldoni apiary (São Paulo, Brazil) and solubilized in saline 0,9% with 5 minute-sonication and heavy agitation in vortex. Following agitation, the solution was collected and diluted at test doses (100, 150, and 200 mg/kg of animal).

### 2.3. Experimental Protocols

Mice were randomly assigned into five groups (*n* = 10): noncolitic saline, negative control group TNBS, in which none received royal jelly and the other 3 groups received, orally, daily for 19 days, 100, 150, and 200 mg/kg of royal jelly (suspended in saline 10 mL/kg). Both saline and TNBS groups were given 10 mL/kg of saline daily. Two weeks after pretreatment started, mice from the TNBS and RJ pretreated groups (100, 150, and 200 mg/kg) (Figure 5) were rendered colitis by the method originally described by Morris et al. [18]. Briefly, they were anaesthetized with halothane and given 10 mg of 2,4,6-trinitrobenzenesulfonic acid (TNBS) dissolved in 0.1 mL of 50% ethanol (v/v) through a Teflon cannula inserted 4 cm through the anus. Mice from the noncolitic group were administered intracolonically 0.25 mL of phosphate-buffered saline instead of TNBS. Behavior, body weight, and stool consistency were recorded daily throughout the experiment. All mice were killed by cervical dislocation 4 days after induction of colitis and the colon was removed and placed on cold plate and longitudinally cut into two slices.

### 2.4. Macroscopic Features of Colitis

The severity of colon damage was macroscopically assessed using the criteria previously established for TNBS-induced colitis [19]. A score ranging from 0 to 10 was employed, as follows: 0, no damage; 1, hyperaemia without ulcers; 2, hyperaemia and wall thickening without ulcers; 3, one ulceration site without wall thickening; 4, two or more ulceration sites; 5, 0.5 cm extension of inflammation or major damage; and 6–10, 1 cm extension of inflammation or severe damage. The score was increased by 1 for every 0.5 cm of damage up to a maximal score of 10; by 0 or 1 for absence or presence of diarrhea and 0, 1, or 2 for absence, presence of mild or severe adhesion, respectively.

### 2.5. Histological Features of Colitis

The colon was dissected and fixed with 4% paraformaldehyde (Merck, Darmstadt, Germany) in 0.1 M phosphate-buffered saline (PBS; pH 7.4) for 24 h at 4°C. The tissues of three animals per experimental group were dehydrated in graded concentrations of alcohol, embedded in historesin (Leica Microsystems, Heidelberg, Germany) and sectioned transversely at a width of 2 *μ*m. The resulting serial sections were mounted on slides, stained with hematoxylin and Floxin and imaged using a Nikon Eclipse E800 light microscope. The images were then qualitatively examined by histopathologists in double-blind experiment. The qualitative histopathological analysis focused on changes of the epithelium integrity, oedema, lymphocytic infiltration, cytoplasmic vacuolization, and necrosis.

### 2.6. Glutathione Level Determination (GSH)

GSH levels of colonic tissue of animals were determined by Ellman's reaction using 5′5′-dithiobis-2-nitrobenzoic acid (DTNB) as described by Anderson [20]. The intensity of the yellow colour was read at 412 nm.

### 2.7. Glutathione Peroxidase Activity (GSH-Px)

GSH-Px activity was quantified by following the decrease in absorbance at 365 nm induced by 0.25 mM H_2_O_2_ in the presence of reduced glutathione (10 mM), NADPH, (4 mM), and 1 U enzymatic activity of GSH-Px [21].

### 2.8. Western Blotting Analyses

Frozen colon samples were homogenized in 1 mL of cold buffer containing phosphate buffer (PB) 0.1 M, pH 7.4, and protease inhibitor cocktail 1% (Sigma-Aldrich P-8340). Homogenates were centrifuged (12,000 ×g, 45 min, 4°C) and the supernatants were collected and stored at −80°C. Different centrifugation times and buffers were used for the cytosolic (14,000 ×g, 45 min, 4°C), membrane (14,000 ×g, 45 min, 4°C), and nuclear extracts (15,000 ×g, 60 min, 4°C) respectively, according to Helenius et al. (1996) with modifications [22]. Protein concentration of the homogenate was determined following Bradford's colorimetric method Bradford [23]. Then, samples were treated with Laemmli buffer (PB 0.5 M, pH 6.8; glycerol, sodium dodecyl sulfate (SDS) 10%, bromophenol 0.1%, and *β*-mercaptoethanol) [24] in a 1 : 1 proportion. Equal amounts of protein from samples (70 *μ*g) were separated on 8% acrylamide gel by sodium dodecyl sulfate polyacrylamide gel electrophoresis. In the next step, proteins were electrophoretically transferred onto a nitrocellulose membrane and incubated with specific primary antibodies: COX-2 (160126) (Cayman Chemical, USA) at 1 : 500 dilution and NF-*κ*B (MAB 2697) (R&D Systems, USA) at 1 : 2000. Each membrane was washed three times for 10 min and incubated with HRP-Goat Anti-Rabbit (Invitrogen 656120) (COX-2, diluted at 1 : 5000). To prove equal loading, the blots were analyzed with standard protein Ponceau dye [24]. Immunodetection was performed using enhanced chemiluminescence light-detecting kit (SuperSignal West Femto Chemiluminescent Substrate, Pierce, IL, USA). Densitometric data were performed with G-BOX, Syngene, following normalization to the control (Ponceau) by GeneSys software.

### 2.9. Statistical Analysis

Results were expressed as mean ± standard error of means (SEM). The statistical significance of each test group in relation to the control was calculated using ANOVA followed by Dunnett's *t*-test.

## 3. Results

### 3.1. Macroscopic Evaluation of Colitis

**Figure 1:**
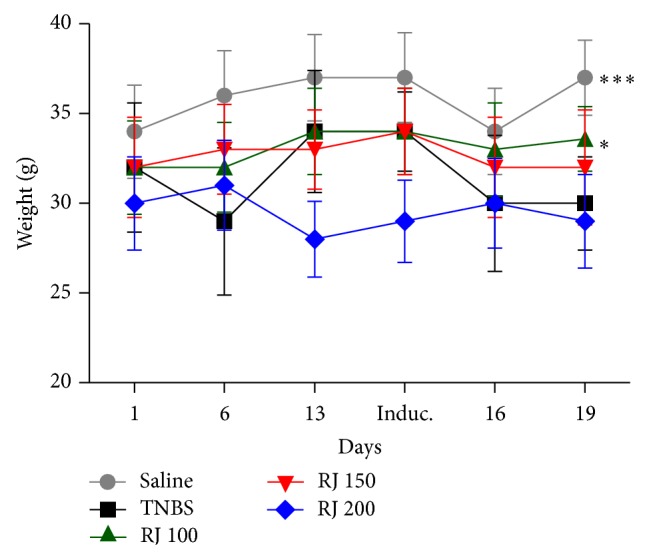
Weight means of the 5 different groups on days 1, 6, and 13 (before induction) and 16 and 19 (after induction). Data is presented as mean ± SEM. Two-way ANOVA followed by Bonferroni's *t*-test. ^*^ *P* < 0.05, ^***^ *P* < 0.001 are significantly different from TNBS group.

Mice subjected to royal jelly pretreatment showed an overall lower impact of TNBS-induced colonic damage compared with the TNBS control group. Macroscopic inspection showed evidence of severe colonic mucosal damage, with oedema, deep ulcerations, and hemorrhage in TNBS group. The doses of 100 and 150 mg/kg significantly diminished the damage provoked by the hapten (*P* < 0.05) (Table 1). When evaluating the adhesion parameter only, the dose of 100 mg/kg was capable of preventing its aggravation. The treated mice with RJ (100 and 150 mg/kg) showed a faster weight recovery than those of the TNBS group (Figure 1). However, mice from the TNBS control group showed a lower weight recovery throughout the experiment in comparison with the mice from the noncolitic group (*P* < 0.05). All the other experiments were performed using only the lowest effective dose.

### 3.2. Histological Evaluation of Colitis

**Figure 2:**
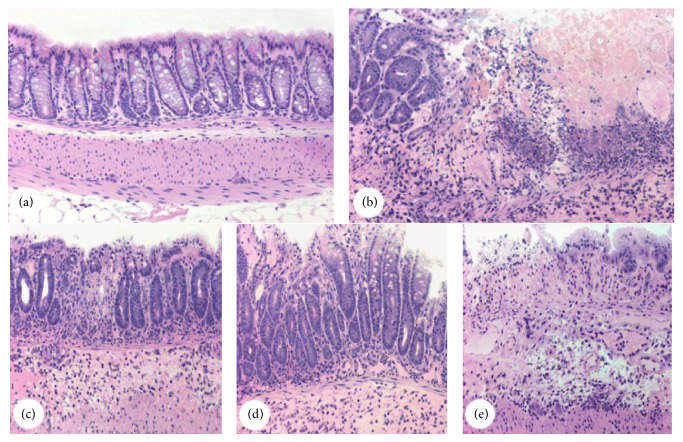
Representative light microscopy of colons from both colitic and noncolitic groups. Micrographs of the groups saline (a), TNBS (b), and RJ 100, RJ 150, and RJ 200 receiving both TNBS and royal jelly (c), (d), and (e), respectively. Hematoxylin and Floxin staining. Micrographs were taken using 10x objective lens. Each image is representative of 3 animals.

The effective induction of TNBS-induced inflammation was corroborated by the loss of epithelium integrity, oedema, and inflammatory infiltrate as shown in Figures 2(a)–2(e).  Figure 2 summarizes the tissue damage from all groups, colitic and noncolitic (Saline, TNBS, RJ 100, 150, and 200 mg/kg). Qualitatively, the noncolitic group saline showed a normal mucosa of the colon with an intact simple columnar epithelium and numerous goblet cells. The crypts of Lieberkühn are straight and unbranched and lined largely with goblet cells. The appearance of the lamina propria is consisted by soft connective tissue, leukocytes, and the muscularis mucosae. Next, the submucosa of this group is made of irregular connective and adipose tissue, numerous blood vessels, and several ganglion cells and nerves of the submucosal plexus.

However, the TNBS-colitic control group showed an extensively loss of epithelium integrity (ulceration) and crypts, loss of goblet cells, and no definite mucosal lining as well as a great number of inflammatory cells that might indicate necrosis. The RJ 100 mg/kg group showed a decreased number of goblet cells, moderate cell infiltrate, mainly neutrophils, and a low intensity oedema when compared with TNBS colitic group. The RJ 150 mg/kg group showed as many goblet cells as noncolitic group saline and an apparently better epithelium integrity; on the other hand, the number of inflammatory cells is qualitatively higher when compared to RJ 100 group. The RJ 200 mg/kg group showed a massive infiltrate and no delimitations of either the mucosal lining or epithelium, similarly to the TNBS control group.

### 3.3. Glutathione Level Determination (GSH)

The GSH levels in the noncolitic group were high and after TNBS colitis induction the GSH levels drastically reduced; on the other hand, RJ 100 mg/kg was capable of preventing the decrease in the levels of GSH after colitis induction. Interestingly, the protection of GSH levels exerted by RJ 100 mg/kg was similar to those of the noncolitic group (Table 2).

### 3.4. Glutathione Peroxidase Activity (GSH-Px)

The noncolitic (saline) and colitic (TNBS) groups were the same regarding GSH-Px activity. RJ at the doses of 100 mg/kg was able to increase the activity of GSH-Px (Table 2).

### 3.5. Western Blotting Analyses

**Figure 3:**
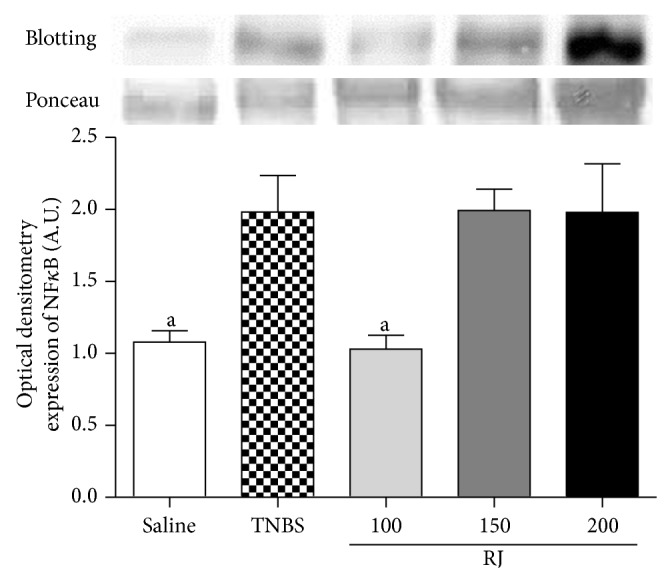
Representative Western blot analysis of analysis of NF-*κ*B. Densitometric data were studied following normalization to the control (Ponceau). The results are representative of three experiments performed on different samples and data are expressed as mean ± SEM. ANOVA followed by Dunnett's *t*-test. ^a^ *P* < 0.05 is significantly different from TNBS group.

**Figure 4:**
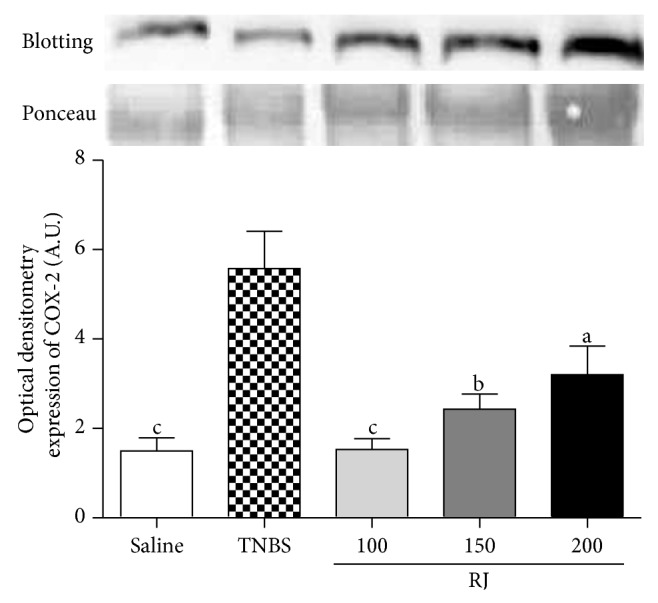
Representative Western blot analysis of COX-2 proteins. Densitometric data were studied following normalization to the control (Ponceau). The results are representative of three experiments performed on different samples and data are expressed as mean ± SEM. ANOVA followed by Dunnett's *t*-test. ^a^ *P* < 0.05, ^b^ *P* < 0.01, and ^c^ *P* < 0.001 are significantly different from TNBS.

**Figure 5:**
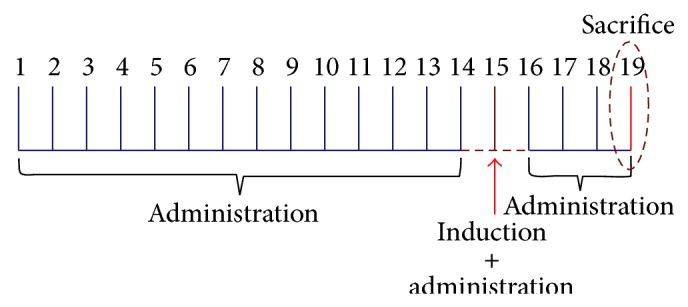
Pretreatment with Royal Jelly (100, 150, and 200 mg/kg) for 14 consecutive days followed by the administration of TNBS (induction of colitis) on day 15. The following 3 days (16, 17, and 18), animals received RJ (100, 150, and 200 mg/kg) and were sacrificed on day 19.

NF-*κ*B and COX-2 from colonic mucosa were measured by western blotting (Figures 3 and 4). The levels of expression of NF-*κ*B p65 were detected in low quantity in nuclei of normal mucosa whereas a high expression of nuclear factor appeared in colon mucosa from control TNBS colitic group. Nonetheless, upon pretreatment with RJ 100 mg/kg, the expression of NF-*κ*B p65 was kept at lower levels than TNBS group (Figure 3). As shown in Figure 4, exposure of colon to TNBS caused strong expression of COX-2; on the other hand, RJ at 100 mg/kg induced downregulation of COX-2 when compared with TNBS group (*P* < 0.001).

## 4. Discussion

Ulcerative colitis (UC) and Crohn's disease (CD) are the two major forms of inflammatory bowel disease (IBD); both diseases share common features, despite differing in the etiology. These two forms of IBD are characterized by an exacerbated immune response towards enteric commensal microbial population [25]. The symptoms are also similar: abdominal pain, severe diarrhea, bloody feces, and consequent anaemia and weight loss. Some patients may lose up to 20% of the total weight in very short periods in the acute phase of the disease, which can be observed in animal models of experimental colitis. Animal models of intestinal inflammation are indispensable for our understanding of the pathogenesis of CD and UC. These models are used to evaluate new anti-inflammatory strategies. One of the most widely used models is colitis induced by the hapten TNBS. It is thought that this model resembles Crohn's disease because of the resulting mucosal inflammation mediated by a Th1 response [26].

We have demonstrated that all colitic groups lost weight after TNBS induction and that the RJ 100 mg/kg group restarted weight gain more quickly than those of the TNBS-colitic group. One must consider that this parameter alone does not indicate, by itself, any efficacy of the pretreatment; however, it gives us an idea of the general state of the animal. The weight loss can be partially explained by the reduction in chow consumption (data not shown) and water intake due to the damage in the colon, which, in turn, causes pain and intense diarrhea. The macroscopic evaluation is a very important tool when assessing experimental TNBS-induced colitis since it considers the size of the ulceration; hence, we can evaluate how damaged the tissue remains [19]. In the TNBS-colitic group, the lesion area was large and showed extensive tissue thickening. RJ 100 mg/kg was effective in protecting the mucosa and keeping the lesion smaller than that of the TNBS group and also presented better macroscopic scores (Table 1). The macroscopic score presented by the dose of 200 mg/kg was not different from the TNBS one. Moreover, it is already known that the royal jelly may exert toxic effects if consumed in high doses or quantities [7–9]; some studies have already demonstrated that animals receiving high amounts of royal jelly presented high death rate due to its toxic effects, which was corroborated by our results. The mechanisms involving its toxicity are still not entirely elucidated, although one of the reasons may be likely to happen due to the presence of polyphenolic compounds in the RJ, including flavonoids [27]. Flavonoids present different biological activities such as antioxidant activity. There is evidence that this compound presents a dual effect, antioxidant or prooxidant, intimately depending on its concentration [28]. It is likely that the dose of 200 mg/kg is exerting a prooxidant effect.

Many aspects are involved in the inflammatory process, such as the oxygen reactive species (ROS), which have already been demonstrated to modulate the immune response. The respiration process is mandatory for the life of aerobic organisms, but it can be harmful due to the formation of ROS. Despite being considered foes, they play very important roles in living organisms. These molecules may exert an antibacterial function through protein, DNA, and lipid oxidation: on the other hand, when they are over produced, they become a threat to cells, due to their great oxidizing capacity [28]. There are extensive data showing that ROS are important players in the pathogenesis of IBD. Increased production of ROS harms the integrity of the epithelial cells, through an initial inflammatory response. In order to protect tissues against ROS-triggered injuries, all cells have antioxidant enzymes, including glutathione peroxidase (GSH-Px) and radical scavengers such as sulfhydryl compounds GSH [11].

GSH has already been shown to have its level diminished in experimental colitis [12]. This is part of the first line of defense against oxidative stress and the pretreatment with RJ 100 mg/kg was capable of increasing the levels of GSH (Table 2). These data suggest that RJ has the capacity of protecting intestinal mucosa from injuries, probably by protecting the depletion of this antioxidant barrier. These results are in accordance with previous studies showing that the RJ exerts a free radical scavenging activity [10], therefore ameliorating the ongoing inflammation.

Recent reports show that the cytosolic GSH-Px activity in rat colon tissues is altered in response to oxidative stress [29]. GSH-Px is an antioxidant enzyme that helps scavenging and inactivating H_2_O_2_, thereby protecting tissues from deleterious damage caused by the peroxide [30].

The activity of GSH-Px appeared reduced in TNBS-colitic groups (Table 2); this apparently paradox can be explained by the GSH and H_2_O_2_ dependence on the activity of the GSH-Px. For the TNBS group, the limiting factor is the level of GSH (very low); on the other hand, for the non-colitic saline group, the limiting factor is the H_2_O_2_, that is not augmented, since there is no oxidative stress going on. In both cases, The RJ 100 mg/kg was capable of increasing the level of GSH as well as the activity of GSH-Px, demonstrating its antioxidant property (Table 2).

Evidences indicate that NF-*κ*B, a ubiquitously expressed, proinflammatory eukaryotic transcription factor is important in the regulation of a broad spectrum of genes in many physiological and pathological events [31]. NF-*κ*B p65 protein is increased in the colonic biopsies of patients with active IBD and a direct correlation with severity was observed with their levels [31]. It has also been reported that the expression of NF-*κ*B is augmented in experimental IBD [29]. Due to the importance of NF-*κ*B, not rarely, researchers make huge efforts in understanding its biology for potential pharmacological uses. In our experimental model, the NF-*κ*B expression was shown to be augmented in the TNBS group, corroborating with reported data [30]. We have also demonstrated that an increase in the colonic level of NF-*κ*B was inhibited by the pretreatment with RJ 100 mg/kg when compared with TNBS group (Figure 4). This NF-*κ*B decreased level is an important parameter in the protection of the gut against chronic TNBS-induced intestinal inflammation model [32].

Cyclooxygenase-2 (COX-2) is one of various inflammatory mediators regulated by NF-*κ*B. The induced form of COX enzyme is involved in numerous physiological responses, mainly inflammation, where they catalyze the synthesis of prostaglandins (PGs) from arachidonic acid [33]. Prostaglandin E_2_ (PGE_2_) is one of the most important biologically active prostanoids found throughout the gastrointestinal tract. Despite the fact that PGE_2_ regulates many physiological functions of the digestive tract, including mucosal protection, gastrointestinal secretion, and motility, it is also implicated in the pathophysiology of IBD [34]. COX-2 is an inducible inflammatory enzyme induced by growth factors, proinflammatory cytokines, tumor promoters, and bacterial toxins and is increased in TNBS-induced colitis [32]. There is sufficient data to support that the inhibition of this enzyme is beneficial during TNBS-induced colitis [35]. Our results showed that the TNBS group showed an increased expression of COX-2 compared with the noncolitic group and that the dose of RJ 100 mg/kg prevented the overexpression of COX-2, when compared with TNBS group. The expression of COX-2 is regulated by the NF-*κ*B and thus, its inhibition may lead to a decrease in the expression of COX-2. Karaca et al. [17] have reported that RJ was capable of reducing the colonic mast cell infiltration, which may stimulate the production of COX-2 as well [36]. These data together (COX-2 inhibition, NF-*κ*B inhibition, and that presented by Karaca et al. [17]) accredits RJ as a potential candidate for the protection against IBD.

## 5. Conclusion

TNBS-induced colitis is a widely accepted model for IBD studies, mainly UC and CD. According to the results shown and the data published, RJ remains as a prominent nutrient. We demonstrated that oral pretreatment with RJ may be effective in protecting the mucosa of mice in TNBS-induced colitis, partly explained by its antioxidant property, which consequently prevents proinflammatory mediators from acutely increasing. It is important, nevertheless, to state that more comprehensive studies need to be carried out in order to evaluate more parameters involved in different models of experimental colitis and that the doses of royal jelly intake must be cautious mainly due to the adverse effects caused by high doses.

## Figures and Tables

**Table 1:** Effect of royal jelly on the disease activity index of mice undergoing TNBS-induced colitis.

Group	Macroscopic assessment	Adhesion	Diarrhea
Noncolitic (saline)	0 ± 0^b^	0 ± 0^b^	0 ± 0^b^
Colitic (TNBS)	4.5 ± 0.42	1.5 ± 0.54	0.71 ± 0.48
RJ 100 mg/kg	2.56 ± 0.29^a^	0.44 ± 0.72^a^	0.67 ± 0.5
RJ 150 mg/kg	2.67 ± 0.236^a^	0.625 ± 0.91	0.62 ± 0.51
RJ 200 mg/kg	3.88 ± 0.515	1.38 ± 0.91	0.750 ± 0.46

Results are presented as mean ± SEM. ANOVA followed by Dunnett's *t*-test. ^a^ *P* < 0.05 and ^b^ *P* < 0.001 are significantly different from colitic nontreated group.

**Table 2:** Effect of royal jelly on colonic GSH level and GSH-Px and GR activities in TNBS-induced colitis in mice.

Group	Colon GSH levels (*μ*mol/mg of protein)	Colon GSH-Px activity (nmol/min/mg protein)
Noncolitic (saline)	25.1 ± 5.91^a^	5.0 ± 0.28
Colitic (TNBS)	8.6 ± 1.10	4.5 ± 0.43
RJ 100 mg/kg	22.8 ± 3.11^a^	11.8 ± 3.18^a^
RJ 150 mg/kg	12.6 ± 3.06	11.0 ± 0.8
RJ 200 mg/kg	5.62 ± 2.56	9.0 ± 0.7

Results are presented as mean ± SEM. ANOVA followed by Dunnett's *t*-test. ^a^ *P* < 0.05 is significantly different from TNBS group.
